# The Law Compelling the Teaching of Physiology and Hygiene in the Public Schools1Discussion before the buffalo Academy of Medicine, September 10, 1895.

**Published:** 1895-11

**Authors:** H. R. Hopkins

**Affiliations:** Professor of hygiene in the University of Buffalo. 433 Franklin Street


### BUFFALO MEDICAL JOURNAL.

Vol. XXXV. NOVEMBER, 1895. No. 4.

### Original Communications.

### THE LAW COMPELLING THE TEACHING OF PHYSIOLOGY AND HYGIENE IN THE PUBLIC SCHOOLS.[1]

WITH PARTICULAR REFERENCE TO ALCOHOLICS AND NARCOTICS.

By H. R. HOPKINS, M. D.,

Professor of hygiene in the University of Buffalo.

YOU will excuse a single observation on the reason why the officers of the Academy thought it wise to put upon its program this evening's discussion. We do not need to go far to find the reason, and it may not be out of place to take a moment of the few allotted to me, to state why this and similar discussions may well engage the time and the best efforts of our members.

The work of the medical profession is honorable at all times, and when well done is always an important factor in the well-being of the community. The highest honor and the weightiest responsibility of our profession is when questions of public health or public morals are up for general discussion, and the time comes for hearing what the doctors from their particular knowledge and experience have to say. There can be no more important topic to any people than that of a right knowledge of physiology and hygiene, and there is no more potent factor at work in the production of suffering and disease than the abuse of alcoholics and narcotics.

This question is now before the people of this state in the shape of a law passed by the last legislature, compelling the teaching of physiology and hygiene, with particular reference to the action of alcoholics and narcotics. It is a question upon which the medical profession has singular knowledge and experience, it is a question which the state may not intelligently settle until the medical profession has spoken, it is a question of great inherent

1. Discussion before the Buffalo Academy of Medicine, September 10, 1895.

difficulty and complexity—touching with its two hands the heart of our educational system, and the most sensitive and important nerves of the body politic,—it is a question in which great popular ignorance and greater popular prejudice make a wrong conclusion particularly easy, and it is a question demanding careful and many-sided study and a right conclusion by every interest of public health and public morality; therefore we may safely conclude that the Academy may profitably discuss this question at this time.

In the year 1884, our legislature enacted a law containing the following provision :

Provision shall be made by the proper local school authorities for instructing all pupils in all schools supported by public money, or under state control, in physiology and hygiene.

If the promoters of this measure had stopped at that point they would have been judged not only earnest and sincere in this agitation, but what is more important, wise in their methods; but they go on and add the words, "with special reference to the effects of alcoholic drinks, stimulants and narcotics upon the human system."

This law is in operation for ten years and apparently does not produce the desired and expected results and in the present year the same people succeed in passing important amendments. The law of 1895 may be said to contain the fully developed thought of the agitators of this matter—a party that call themselves the scientific temperance party—and with the passage of the law of 1895 the matter for the first time attracted general public notice. The law is short and I give it entire :

### LAW OF 1895.—Chapter 1041.

AN ACT

To amend the consolidated school law providing for the study of the nature and effects of alcoholic drinks and other narcotics, in connection with physiology and hygiene in the public schools, approved June 15th.

*The People of the State of New York, represented in Senate and Assembly, do enact as follows :*

Section 1. Sections nineteen and twenty of article fifteen of the consolidated school law are amended to read as follows :

§ 19. The nature of alcoholic drinks and other narcotics and their effects on the human system shall be taught in connection with the various divisions of physiology and hygiene as thoroughly as are other branches for not less than four lessons a week for ten or more weeks in each year in all grades below the second year of the high school in all schools under state control, or supported wholly or in part by public money, and also in all schools connected with reformatory institutions. All pupils must continue such study till they have passed satisfactorily the required primary, intermediate or high school test in the same, according to their respective grades. All regents' examinations in physiology and hygiene shall include a due proportion of questions on the nature of alcoholic drinks, tobacco and other narcotics, and their effects on the human system. The local school authorities shall provide facilities and definite time and place for this branch of the regular course of study. All pupils who can read shall study this subject from suitable text-books, but pupils unable to read shall be instructed in it orally by teachers using text-books adapted for such instruction as a guide and standard, and these text-books shall be graded to the capacities of primary, intermediate and high school pupils. For students below high school grade they shall give at least one-fifth their space, and for students of high school grade shall give not less than twenty pages to the nature and effects of alcoholic drinks and other narcotics, but pages on this subject in a separate chapter at the end of the book shall not be counted in meeting the minimum. No text-book on physiology not conforming to this act shall be used in the public schools except so long as may be necessary to fulfill the conditions of any contract existing on the passage of this act.

§ 20. In all normal schools, teachers' training classes and teachers' institutes, adequate time and attention shall be given to instruction in the best methods of teaching this branch, and no teacher shall be licensed who has not passed a satisfactory examination in the subject, and the best methods of teaching it. No state school money shall be paid for the benefit of any district, city, normal or other school herein mentioned, until the officer or board having jurisdiction and supervision of such school has filed with the officer whose duty it is in each case to disburse the state school money for such school an affidavit made by such officer, or by the president or secretary of such board, that he has made thorough investigation as to the facts and that to the best of his knowledge, information and belief all the provisions of this act have been faithfully complied with during the preceding school year.

§ 2. This act shall take effect August 1, 1895.

You will observe that the thought and purpose of the promoters of the so-called scientific temperance have unfolded not a little between the years 1884 and 1895. What that purpose really is we may judge by looking at these two laws and at the text-book and teaching these laws would compel. At the first we were to be taught the effects of alcoholic drinks, stimulants and narcotics, but later the demand is that in addition to this, we shall be taught the

"nature of alcoholic drinks and other narcotics." If this means anything, it means that we are to be taught chemistry—both inorganic and organic, or at least so much of chemistry as will put the pupil abreast of the knowledge of the various alcohols, alkaloids, ethers and other chemical substances found in "alcoholic drinks and other narcotics." How densely ignorant our promoters of scientific temperance are of their own subject as also of educational matters, we get a hint when we remember that the law of 1895 demands that this teaching be done to children who have not yet learned to read, "but pupils unable to read shall be instructed orally." Before proceeding to state some of the objections to this law, objections which, in my mind, demand its early repeal, let me call to your attention that these alcoholic and narcotic matters are to be taught our children for at least four times per week and for at least ten weeks per year, and this upon penalty of withdrawal of state support from any school failing so to teach.

In presenting the objections which come to my mind, please remember that the speaker is making an extra effort to use such moderate and balanced language as befits the dignity of the occasion and the serious importance of the issue involved. His inclination is almost overwhelming to take refuge in invective and execration. He does not remember ever to have met a more conspicuous example of proper motives and improper and impossible methods than in this campaign of scientific temperance.

My first objection to this law relates to the manner of its passage and is to the effect that it presumes to invade the domain of two distinctly learned professions, our educators and our guardians of public health, and to dictate to both how they shall think and how they shall act and that without conference or consultation with either. The medical profession, as the same is known to the state, is represented by three state organisations, each holding annual meetings at Albany during the session of the legislature which passed this bill, and so far as I have heard this matter was not submitted to either, or to our state board of health. Our educational system is ably represented at Albany by the regents of the university and by the state superintendent of public instruction and by the state teachers' association. The principles of this law strike at the very heart of the teachers' art. Any of these could give our promoters of scientific temperance advice of the greatest value, but such advice was not sought.

Omissions like these are sufficient ground for able-bodied and ugly suspicions as to the structural soundness of this law.

Let us turn aside for a moment and see what light the text-books, advised by our promoters of temperance of the scientific order, throw upon this law or upon their motives and purposes.

On enquiry at our high school I learned that the following was in use in our schools: "Hygienic Physiology, with special reference to the use of alcoholic drinks and narcotics, adapted from the Fourteen Weeks in Human Physiology, by Joel Dorman Steele, Ph. D.; edited and endorsed for the use of schools (in accordance with the recent legislation upon this subject), by the department of scientific temperance instruction, of the Women's Christian Temperance Union, of the United States, under the direction of Mrs. Mary H. Hunt, superintendent." This is a book of over 275 pages, is copiously illustrated with numerous colored full page drawings, and presumes to teach human anatomy and physiology. You will allow me to observe right here that I have always had the conviction that anatomy and physiology are not suitable subjects for teaching in our public schools and that this conviction was deepened by the perusal of the text-book now under consideration. Of this it must suffice to remark that the figures presented by drawings and text do not belong to the human species or to any other family of the animal kingdom.

We will now try and see what our friends of scientific temperance would have our children taught:

Page 128.—Alcoholic Drinks and Narcotics.—Place on the web of the frog's foot a drop of dilute spirit. The blood-vessels immediately expand, an effect known as "vascular enlargement." Channels before unseen open and the blood-discs fly along at a brisker rate. Next, touch the membrane with a drop of pure spirit. The blood channels quickly contract; the cells slacken their speed and, finally, all motion ceases. The flesh shrivels up and dies. The circulation thus stopped is stopped forever. The part affected will in time slough off. Alcohol has killed it. The influence of alcohol upon the human system is similar. Alcohol is a poison.

Page 133.—Wherever the alcoholised blood goes through the body, it bathes the delicate cells with an irritating, narcotic poison, instead of a bland, nutritious substance.

Page 163.—Is alcohol a food? To answer this question, let us make a comparison. If you receive into your stomach a piece of bread or beef, nature welcomes its presence. The juices of the system at once take hold of it, dissolve it and transform it for the uses of the body. . . . . .

If, on the other hand, you take into your stomach a little alcohol, it receives no such welcome. Nature treats it as a poison and seeks to rid herself of the intruder as soon as possible. The juices of the system will flow from every pore to dilute and weaken it, and to prevent its shriveling up the delicate membranes with which it comes in contact. The veins will take it up and bear it rapidly through the system. Every organ of elimination, all the scavengers of the body—the lungs, the kidneys, the perspiration glands—at once set to work to throw off the enemy. So surely is this the case, that the breath of a person who has drunk only a single glass of the lightest beer will betray the fact. . . . .

Alcohol, then, is not, like bread or beef, taken hold of, broken up by the mysterious process of digestion and used by the body. "It cannot, therefore, be regarded as an aliment" or food.—(Flint). "Beer, wine and spirits," says Leibig, "contain no element capable of entering into the composition of the blood or the muscular fiber." "That alcohol is incapable of forming any part of the body," remarks Cameron, "is admitted by all physiologists. It cannot be converted into brain, nerve, muscle or blood."

I will not detain you with further quotations from this text-book of hygienic physiology, but observe that this matter is to be taught to our innocent and helpless children before they have learned to read and that in the name of science and by the express command of the state. You will also remember that this book and its peculiar teaching is the offspring of the laws demanding the teaching of physiology in our public schools and we will make no mistake when we construe this law and the motives of its promoters in the light of their statements, found in their text-book, as to the effects on the human system of alcoholic drinks.

Before trusting ourselves to characterise these statements let us steady our minds with a few of the sober, balanced and judicial utterances of scientific men, made in the interests of public health:

Reference Hand-book of Medical Sciences, page 102. ALCOHOL.—PHYSIOLOGICAL ACTION.—*Stomach and Intestinal Tract.*—The action of this agent upon the stomach and intestinal tract has been one of the bones of contention between the advocates of teetotalism and those who, knowing its virtues, can utilise them. To incorporate the arguments of both sides would be foreign to the scope of this article which is to give the present status of scientific opinion.

Small quantities of alcohol, properly diluted, taken into the stomach, produce an agreeable sensation of warmth which soon diffuses itself over the entire body. It is quickly absorbed. . . . .

*Brain and Nervous System.*—The primary effect of alcohol (in small quantities) on the nervous system is a stimulation of the functional activity of the brain. This is a result chiefly of a direct stimulation of the alcohol upon the nervous tissue through the increased force of the heart-beat. Its increased frequency and the greater activity of the entire bodily functions undoubtedly assist its local action.

A sense of well-being pervades the body, a greater activity of intellection, increased volubility and a general exhilaration result, which, enduring for a time, are followed by no depression. . . . .

*Excretion.*—Experimenters all agree in this, that not more than 16 per cent. of the alcohol taken can be found in the excreta. The greater portion disappears in the system. As to its mode of destruction, nothing is positively known. . . . If it is destroyed by oxidation, as we have reason to believe, carbonic acid and water, both normal constituents of the blood, would be the final products and could not be identified as derived from alcohol. . . . .

*Administration.*—The physical constitution of the patient, together with the state of the health and the result to be acquired, must form the guide to the proper selection and doses. The carefully conducted experiments of Dujardin-Beaumetz, Richardson and others, agree that one gramme (15 grains) of absolute alcohol to every kilo (2 lbs.) of body-weight, is about the daily limit that can be assimilated by the healthy adult without disturbance of digestion or other injurious consequences. It is true, however, that patients exhausted by the continued fevers can absorb amounts far exceeding the normal limits without injury, in fact, with benefit. . . . .

It may be generally stated that the stronger wines are indicated by the weakened conditions occasioned by the long continued fevers, chronic suppurative processes and anemia from frequent hemorrhages and for convalescents generally. . . . .

Brandies, whiskies, etc. (of good quality), are indicated, undiluted, in cases of sudden weakening of the heart's action. Given after a full meal they certainly aid its digestion. This group of alcoholic drinks, when not abused, take the place, with the poor, of the costly condiments of the rich, improving the appetite and aiding digestion. Diluted, they can be used where wines are indicated, but not as efficiently. . . . .

The beers, ales and porters are valuable because of the nutritious material they contain. They are readily assimilated and are pleasant to the taste, and the bitter principles contained in them, together with the alcohol, cause an increased flow of gastric juice. They are, therefore, prescribed with food as a dietary measure. The diastase which exists in the beer is present in sufficient quantity to aid in the conversion of the starchy foods.

Their effect upon the brain is not so pleasant as that of wine due

(according to Rossbach) to the oil of hops, which resembles in physiological action, oil of turpentine. They are desirable for those who cannot stand the cerebral effects of wines.

Parkes Hygiene, page 325.—BEVERAGES AND CONDIMENTS.—*Conclusion as to the Use of Alcohol.*—It does not appear possible at present to condemn alcohol altogether as an article of diet in health; or to prove that it is invariably hurtful as some have attempted to do. . . .

As a matter of public health, it is most important that the medical profession should throw its great influence into the scale of moderation; should explain the limit of the useful power, and show how easily the line is passed which carries us from the region of safety into danger, when alcohol is taken as a common article of food. . . .

*Dietetic uses of Alcoholic Beverages.*—In wine there are some albuminous substances, much sugar (in some wines) and other carbo-hydrates and abundant salts. Whether it is that the amount of alcohol is small, or whether the alcohol itself be, in some way, different from that prepared from distillation, or whether the coëxistence of carbo-hydrates and of salts modifies its action, certain it is that the moderate use of wine, which is not too rich in alcohol, does not seem to lead to those profound alterations of the molecular constitution of organs, which follow the use of spirits, even when not taken largely. Considering the large amount of vegetable salts which most wines contain, it may reasonably be supposed that they play no unimportant part in giving dietetic value to wine. . . .

In beer there appears to be four ingredients of importance—namely, the extractive matters and sugar, the bitter matters, the free acids and the alcohol. The first, no doubt, are carbo-hydrates and play the same part in the system as starch and sugar, appropriating the oxygen and saving fat and albuminates from destruction. Hence, one cause of the tendency of persons who drink much beer to get fat. The bitter matters are supposed to be stomachic and tonic, though it may be questioned whether we have not gone too far in this direction, as many of the highest-priced beers contain now little else than alcohol and bitter extract. The action of the free acids is not known, but their amount is not inconsiderable, and they are mostly of the kind which form carbonates in the system and which seem to play so useful a part. The salts, especially potassium and magnesium phosphates are in large amount.

It is evident that in beer we have a beverage which can answer several purposes, viz.: can give a supply of carbo-hydrates of acid, of important salts, and of a bitter tonic (if such be needed) independent of its alcohol. . . .

In moderation it is no doubt well adapted to aid digestion and to lessen to some extent the elimination of fat.

Text-Book of Hygiene. Third edition. Rohé. Page 119. *Beverages Containing Alcohol.*—Alcohol is not necessary to persons in good health. Probably most persons regardless of their state of health, do better without it. Its habitual use in the form of strong liquors is to be unreservedly condemned. The lighter wines and malt liquors, if obtained pure, may be consumed in moderate quantities without ill effects. Even in these forms, however, the use of alcohol should be discouraged or, perhaps, prohibited in the young. . . .

Beer and its correlatives have considerable dietetic value, owing not merely to the alcohol they contain, but largely to the sugar and acids entering into their composition. When used to excess they often cause a considerable accumulation of fat.

Practical Hygiene. Coplin and Bevan. Page 174. *Food.*—Alcoholic beverages as a food. Yeo, in sympathy with Parkes and others, believes that the minimum amount of alcohol, whether in the form of spirits, wine or beer, which should be taken by a healthy adult, should not exceed one and one-half fluid ounces in the twenty-four hours. In this amount it is believed to be a stimulant to the circulatory, respiratory and nervous system, increasing the appetite and facilitating digestion.

Gentlemen, this is the teaching of the science of medicine upon this subject, and unless we are content to have our text-books the laughing stock of students we will see to it that whatever is taught on the effects of alcoholic drinks upon the human system shall be after this manner.

Regarding the text-book produced by our alleged promoters of scientific temperance, it may be said that there is but slight relation between title and subject matter. Professing to instruct innocent and helpless children upon the effects of alcoholic drinks, it gives a lurid and hysterical dissertation, in language almost vulgar, upon absolute anhydrous alcohol, a substance known only to the chemist and then only as a rare chemical curiosity. As a whole, nothing could be more misleading, misrepresenting and language can scarcely be more immoral.

It is impossible to believe that good can come from such false witness and false teaching, and it needs no very keen insight to foresee that the future public health and morals is seriously menaced by this well-meaning jesuitism. This movement seems to be devised to compel the teaching of the contents of this book to the youths of this state and, regardless of the high motives of its promoters, regardless of who voted for the bill or who signed the law, I do not hesitate to charge that it came from Sheol and is in the handwriting of the father of lies.

But, let us advance from details to principles, and suppose that the so-called promoters of scientific temperance had come with a bill giving the best thought of the most competent educators of the state upon the fundamental question, Can the physiology and hygienics of alcoholics and narcotics be taught in our public schools with advantage? And if this simple question, without errors or blunders, was put to the medical profession, what have we to say? After relieving the question of every embarrassment which the narrowness, the ignorance, the fanaticism of its friends have put upon it, is there sufficient merit in the case to warrant the state in placing upon its statute books the order compelling this teaching?

To which I make answer, without hesitation or reservation, there is not. And this answer is given in the light of the knowledge, experience and responsibility of many years as family physician, as teacher of hygiene, as one proud to be a citizen of the Empire state, and, more than all beside, as the father of a Christian family. To my mind there are three objections to the root principle of this law, either of which, if valid, is reason sufficient for its repeal.

*First.*—That the fundamental conception of this law is an offense against science, in that it proposes, in the name of science, to store the mind with facts of a kind the mind is utterly unable to receive, arrange, digest or assimilate and that the result must be either waste of time and effort on the part of child and teacher, or the lodgement in the mind of the child of a brood of half truths, partial truths, distorted truths, certain to stunt the mind, to deform the mold of thought, to produce a pseudo-scientist, the most helpless, hopeless, intractable, incorrigible citizen with which we may burden the state. The pseudo-scientist is the thorn, the thistle, the mildew, the canker worm, the pest of our time; his breed does not need cultivation.

*Second.*—The thought of this law is an offense against art, in that it violates the principle of form and place and demands that the loathsome facts and secrets of the pathological museum, and the physician's consulting chamber be paraded in our drawing-rooms, class-rooms and nurseries; that innocent minds be made common sewers; that the Kreutzer Sonata and the Heavenly Twins be provided as text-books—recreative and instructive reading for our growing girls and boys. The potential evil of this wholesale poisoning of springs, this widespread destruction of innocence, can neither be overestimated nor overstated.

*Third.*—This law is an offense against religion in thought and in act, in what it assumes and in what it does. It entrenches upon and violates the domain and province of religion, the right to instruct in morals, and proposes to substitute the dicta of the chemist and the physiologist, in the place of the command from Sinai. But it may be urged against this position, that religion has no place in our public school system and that, therefore, there can be no intrusion, that science is at home in her own house and may do as she likes with her own. To which we may observe that there are questions and more particularly those of morality and religion which town-meetings, state legislatures or general governments do not settle.

All of these may have determined that morality and religion shall not be taught in our public schools and the result will be that morality and religion will be taught in our public schools or our schools will cease to teach anything.

I would commend to the most serious attention of our thinking citizens the most significant fact that some forty of our states have made provision by statute to enforce in different ways that monstrous conception, the teaching of a so-called scientific morality. No state, however, has gone as far as New York.

Again, this law offends religion in that it reverses the value of soul and body and would teach a material morality, having as its object the preservation of the body, to the neglect of the more important duty and responsibility of the care of the soul. The preservation of chastity and temperance in a singular degree is the province of religion. In this work her "Thou shalt not" sounds on from age to age—where religion is direct, positive, authoritative—science is hesitating and indefinite. "Ye shall not surely die" is her usual expression.

In consideration of the foregoing it would seem to be the duty of the medical profession to advise in the interest of science, art and religion that it is inexpedient to urge the teaching in our common schools of physiology regarding the effects of alcoholic drinks and narcotics, but should other counsels prevail and after proper consideration of the matter it should still be determined to teach our children of this matter, then, surely, it is the duty of the profession to demand in the interests of morality and decency that what is taught shall be the simple truth.

433 Franklin Street.

